# A de novo transcriptome assembly for the bath sponge *Spongia officinalis*, adjusting for microsymbionts

**DOI:** 10.1186/s13104-019-4843-6

**Published:** 2019-12-18

**Authors:** Tereza Manousaki, Vasiliki Koutsouveli, Jacques Lagnel, Spyridon Kollias, Costas S. Tsigenopoulos, Christos Arvanitidis, Antonios Magoulas, Costas Dounas, Thanos Dailianis

**Affiliations:** 1 Institute of Marine Biology, Biotechnology & Aquaculture, Hellenic Centre for Marine Research, Heraklion, Greece; 2 Life Sciences Department, Natural History Museum of London, London, UK; 3 Institut National de la Recherche Agronomique PACA, UR 1052 GAFL, Montfavet Cedex, France; 4 Norwegian Sequencing Centre, Centre for Ecological and Evolutionary Synthesis, University of Oslo, Oslo, Norway

**Keywords:** Porifera, Marine invertebrate, Prokaryotic symbionts, RNAseq, Heat stress, Bioinformatics

## Abstract

**Objectives:**

We report a transcriptome acquisition for the bath sponge *Spongia officinalis*, a non-model marine organism that hosts rich symbiotic microbial communities. To this end, a pipeline was developed to efficiently separate between bacterial expressed genes from those of eukaryotic origin. The transcriptome was produced to support the assessment of gene expression and, thus, the response of the sponge, to elevated temperatures, replicating conditions currently occurring in its native habitat.

**Data description:**

We describe the assembled transcriptome along with the bioinformatic pipeline used to discriminate between signals of metazoan and prokaryotic origin. The pipeline involves standard read pre-processing steps and incorporates extra analyses to identify and filter prokaryotic reads out of the analysis. The proposed pipeline can be followed to overcome the technical RNASeq problems characteristic for symbiont-rich metazoan organisms with low or non-existent tissue differentiation, such as sponges and cnidarians. At the same time, it can be valuable towards the development of approaches for parallel transcriptomic studies of symbiotic communities and the host.

## Objective

Sponges are organisms with simple body plan, lacking true tissue differentiation [1]. Moreover, they often host rich symbiotic bacterial communities, thus creating complex holobionts [2, 3]. These traits, combined with the diverse nature of the poriferan phylum and their vulnerability to global change makes them ideal case-study species (e.g. [4–6]). Although transcriptomic studies facilitated through NGS can provide sound answers to ecological questions, the lack of a reference genome makes the building a de novo assembly necessary, as for all non-model organisms. This becomes more challenging in sponges, as it is often difficult to discriminate between signals of metazoan and prokaryotic origin [7, 8], thus introducing biases to interpretation.

Here, we constructed the transcriptome of the Mediterranean bath sponge *Spongia officinalis*, an organism that has suffered a substantial decline in the past decades due to the combined impact of harvesting and mass mortalities attributed to extreme climatic events [9, 10]. The acquisition of the transcriptome was used to assess gene expression within a manipulative experiment, where individuals of the sponge were subjected to a gradient of elevated temperatures simulating extreme climatic events currently occurring during the warm season in its native habitats (see Table 1 data file 1 for experimental design). The results of the study are published in [4] and all data files are presented in Table 1.

**Table 1:** Overview of data files/data sets

Label	Name of data file/data set	File types (file extension)	Data repository and identifier (DOI or accession number)
Data file 1	Figure 1—Experimental design	png	Figshare (10.6084/m9.figshare.10001870.v3) [16]
Data file 2	Figure 2—Sponge RNA extraction on gel agarose	png	Figshare (10.6084/m9.figshare.10001870.v3)
Data file 3	Figure 3—Bioinformatic pipeline	png	Figshare (10.6084/m9.figshare.10001870.v3)
Data file 4	Scythe_batch	sh	Figshare (10.6084/m9.figshare.10001870.v3)
Data file 5	PE_sickle_prinseq_trimmomatic_batch	sh	Figshare (10.6084/m9.figshare.10001870.v3)
Data file 6	allreads_trinity	.fasta	Figshare (10.6084/m9.figshare.10001870.v3)
Data file 7	getit_bacteria	.pl	Figshare (10.6084/m9.figshare.10001870.v3)
Data file 8	assembly_nobacteria_pass1	.fasta	Figshare (10.6084/m9.figshare.10001870.v3)
Data file 9	assembly_nobacteria_final	.fasta	Figshare (10.6084/m9.figshare.10001870.v3)
Data file 10	Extract_reads_from_bam	.sh	Figshare (10.6084/m9.figshare.10001870.v3)
Data set 1	All raw reads from the 20 libraries	.fastq.gz	SRA (SRP150632) https://www.ncbi.nlm.nih.gov/sra/?term=SRP150632 [17]

The built transcriptome assembly comprises the only transcriptome reference available for *S. officinalis* and can serve as a baseline for further studies on the species. This transcriptome reference has already been used in studies of different focus (see [11]) indicating the importance of this transcriptome generation in various study fields. The proposed pipeline can be followed to overcome the technical RNASeq problems characteristic for symbiont-rich metazoan organisms with low or non-existent tissue differentiation, such as sponges and cnidarians.

## Data description

Four *S. officinalis* individuals collected from natural populations from the island of Crete, Greece, were reared in closed tanks and experimentally exposed to elevated temperatures approximate an extreme climate event naturally occurring in the sponge’s habitat during summer. The 50 m^3^ rearing tanks contained natural seawater collected from a pristine open-sea area, with temperature and salinity adjusted to reflect typical local conditions for the time of year (24 °C and 39 ppt, respectively). Two experimental tanks were employed, one as control (24 °C) and one as treatment with increasing temperature (up to 30 °C). Five sampling points initiated after 5 days acclimatization in the tanks and over a span of 6 days, resulted in 20 samples. RNA was extracted with TRIZOL (TRIzol™ Reagent, Thermo Fisher Scientific, Cat. number 15596026) following the manufacturer’s protocol. The quality control of the RNA revealed a unique profile. Apart from the expected 28 s, 18 s ribosomal bands two extra bands, possibly of 23 s, 16 s characteristic of the microbial ribosomal RNA, appeared at the agarose gel, which reflected a remarkably large proportion of prokaryotes in the extracted RNA (data file 2). For the library preparation we used the TruSeq Stranded mRNA LT Sample Prep Kit (Illumina, Cat. Number 20020594) and followed the protocol of the manufacturer for sequencing using the shortest possible fragmentation time and applying 13 cycles instead of 15 in the amplification library PCR at the very last step of the protocol. In total, 20 RNA libraries were sequenced on an Illumina HiSeq 2000 platform. Τhe amount of prokaryotic RNA in our extraction urged us to implement extra steps for excluding the prokaryotic sequences from our dataset (data file 3).

Sequencing yielded on average 12,933,232 raw paired reads per library (data set 1). Raw reads were quality controlled using multiple software in a workflow described in [12] and run through bash scripts (data file 4 and 5). The used software included scythe (version 0.994 BETA; https://github.com/vs.buffalo/scythe), sickle (version 1.33; https://github.com/najoshi/sickle), prinseq (version 0.20.4; http://prinseq.sourceforge.net/) and trimmomatic version 0.32 [13]. The quality-controlled data were used to build an initial Trinity (v2.1.1) [14] assembly (data file 6). However, given that a great percentage of sponge transcriptome is comprised of bacterial sequences, we downloaded all bacterial sequences from NCBI (data file 7) and removed all reads (2.2 to 17.6% of the reads of each sample) that were successfully mapped on them using riboPicker (ribopicker-standalone-0.4.3 version; https://sourceforge.net/projects/ribopicker/files/standalone/; command *ribopicker.pl* -*c 47* -*i 75* -*l 40* -*z 3*). Then, we built another assembly with the remaining reads (data file 8). The reconstructed transcripts were then used for a similarity search through NOBLAST [15] against the Swiss-Prot database (e-value: 1.0E−5). Transcripts that had as best hit prokaryotic sequences (17.1% of the assembly) were eliminated leading to the final assembly (data file 9). Their corresponding reads were eliminated from the bam files as well (data file 10) and were excluded from downstream analyses.

## Limitations

The proposed pipeline eliminates effectively most prokaryotic sequences within the sequenced dataset, however, it does not filter out non-sponge eukaryotic sequences that are often present due to existence of symbiotic eukaryotes as well, e.g. fungi and dinoflagellates.

## Data Availability

The data described in this Data note can be freely and openly accessed on figshare (10.6084/m9.figshare.10001870.v3) and SRA (https://www.ncbi.nlm.nih.gov/sra/?term=SRP150632). Please see Table 1 and reference list for details and links to the data.

## Abbreviations

RNASeq: RNA-sequencing the use of next-generation sequencing to assess the presence and quantity of the expressed RNA in a biological sample
NGS: next-generation sequencing
